# Geometric and dosimetric evaluations of atlas-based segmentation methods of MR images in the head and neck region

**DOI:** 10.1088/1361-6560/aacb65

**Published:** 2018-07-11

**Authors:** J P Kieselmann, C P Kamerling, N Burgos, M J Menten, C D Fuller, S Nill, M J Cardoso, U Oelfke

**Affiliations:** 1 Joint Department of Physics, The Institute of Cancer Research and The Royal Marsden NHS Foundation Trust, London, United Kingdom; 2 University College London, Centre for Medical Image Computing, London, United Kingdom; 3 Department of Radiation Oncology, MD Anderson Cancer Center, Houston, TX, United States of America; 4 Inria, Aramis project-team, Institut du Cerveau et de la Moelle épinière, Sorbonne Université, Paris, France; 5 School of Biomedical Engineering and Imaging Sciences, King’s College, London, United Kingdom; jennifer.kieselmann@icr.ac.uk

**Keywords:** automated segmentation, head and neck, treatment planning, MRI

## Abstract

Owing to its excellent soft-tissue contrast, magnetic resonance (MR) imaging has found an increased application in radiation therapy (RT). By harnessing these properties for treatment planning, automated segmentation methods can alleviate the manual workload burden to the clinical workflow.

We investigated atlas-based segmentation methods of organs at risk (OARs) in the head and neck (H&N) region using one approach that selected the most similar atlas from a library of segmented images and two multi-atlas approaches. The latter were based on weighted majority voting and an iterative atlas-fusion approach called STEPS. We built the atlas library from pre-treatment T1-weighted MR images of 12 patients with manual contours of the parotids, spinal cord and mandible, delineated by a clinician. Following a leave-one-out cross-validation strategy, we measured the geometric accuracy by calculating Dice similarity coefficients (DSC), standard and 95% Hausdorff distances (HD and HD95), and the mean surface distance (MSD), whereby the manual contours served as the gold standard. To benchmark the algorithm, we determined the inter-observer variability (IOV) between three observers.

To investigate the dosimetric effect of segmentation inaccuracies, we implemented an auto-planning strategy within the treatment planning system Monaco (Elekta AB, Stockholm, Sweden). For each set of auto-segmented OARs, we generated a plan for a 9-beam step and shoot intensity modulated RT treatment, designed according to our institution’s clinical H&N protocol. Superimposing the dose distributions on the gold standard OARs, we calculated dose differences to OARs caused by delineation differences between auto-segmented and gold standard OARs. We investigated the correlations between geometric and dosimetric differences.

The mean DSC was larger than 0.8 and the mean MSD smaller than 2 mm for the multi-atlas approaches, resulting in a geometric accuracy comparable to previously published results and within the range of the IOV. While dosimetric differences could be as large as 23% of the clinical goal, treatment plans fulfilled all imposed clinical goals for the gold standard OARs. Correlations between geometric and dosimetric measures were low with R^2^  <  0.5.

The geometric accuracy and the ability to achieve clinically acceptable treatment plans indicate the suitability of using atlas-based contours for RT treatment planning purposes. The low correlations between geometric and dosimetric measures suggest that geometric measures alone are not sufficient to predict the dosimetric impact of segmentation inaccuracies on treatment planning for the data utilised in this study.

## 1. Introduction

Magnetic resonance (MR) imaging has found an increased application in image guidance for radiation therapy (RT) owing to its superior soft-tissue contrast and lack of ionising radiation compared to the conventionally used x-ray computed tomography (CT) (Metcalfe *et al* 2013, Dirix *et al* 2014, Lagendijk *et al* 2014). High soft-tissue contrast MR images are used to improve the delineation of volumes of interest (VOIs) on the CT for the treatment planning, as well as for treatment adaptations (Chung *et al* 2004, Emami *et al* 2003, Rasch *et al* 2010). The accurate localisation of all organs at risk (OARs) and radiation targets is necessary when applying sharp dose gradients in the treatment planning. In MR-only treatment workflows, the MR image replaces the conventionally used pre-treatment CT (Nyholm and Jonsson 2014, Köhler *et al* 2015). Treatment planning and dose calculation are solely based on the MR image but are challenging as the required electron density information cannot be derived directly from image intensities. Therefore, methods such as creating synthetic CTs are necessary to provide surrogates for electron densities (Edmund and Nyholm 2017). In-room image guidance can be provided by combined MR imaging and treatment systems (Raaymakers *et al* 2009, Fallone *et al* 2009, Mutic and Dempsey 2014, Liney *et al* 2016). These systems enable the possibility to scan the patient directly prior to or during the treatment and to adapt the radiation delivery according to the updated information on the patient’s anatomy through MR imaging for the same treatment fraction. Clinicians conventionally delineate all VOIs prior to treatment. This is especially tedious for the treatment of head and neck (H&N) cancer patients due to the complex anatomy including many OARs and target volumes. Many of these VOIs are difficult to delineate on a CT and would hence benefit from MR imaging (Schmidt and Payne 2015). Automating the delineation of VOIs would alleviate the enormous workload of manual delineation and reduce inter- and intra-observer variabilities (Vinod *et al* 2016). Numerous studies have investigated CT-based automated delineation of critical structures in the H&N region (Han *et al* 2008, Sims *et al* 2009, Pekar *et al* 2010, Faggiano *et al* 2011, Qazi *et al* 2011, La Macchia *et al* 2012, Daisne and Blumhofer 2013, Fritscher *et al* 2014, Hoang Duc *et al* 2015), yet only a few studies have been conducted on MR images (Yang *et al* 2014, Veeraraghavan *et al* 2015, Wardman *et al* 2016). The most commonly used automated delineation methods are atlas-based (Fritscher *et al* (2014) and references therein).

The performance of auto-segmentation algorithms is commonly evaluated in terms of geometric criteria only. However, in RT it is relevant to quantify the impact of an inaccurate VOI localisation on the planned dose distribution. A few groups have addressed this need and looked at dosimetric differences on CT images in various attempts (Tsuji *et al* 2010, Voet *et al* 2011, Nelms *et al* 2012, Conson *et al* 2014, Beasley *et al* 2016, Eldesoky *et al* 2017). To our knowledge, as yet, no single geometric measure has been observed to be suitable for prediction of the dosimetric outcome. To properly address the dosimetric impact of segmentation inaccuracies in the process of generating treatment plans, we have calculated dose distributions, optimised for the automatically delineated VOIs, and investigated resulting dose differences to the respective gold standard VOIs. Voet *et al* (2011) and Beasley *et al* (2016) also used this approach to investigate the dosimetric differences and their correlations to geometric measures on CT images of H&N cancer patients.

In this study, we propose to investigate the dosimetric impact of auto-generated contours on MR images by establishing a fully automated workflow consisting of

(1) automated atlas-based segmentation of the parotids, the spinal cord and the mandible on MR images of H&N cancer patients
(2) automated treatment planning for any set of VOIs using a template approach
(3) automated geometric and dosimetric evaluation of auto-generated VOIs where manually drawn contours serve as the gold standard reference
(4) benchmarking the auto-segmentation algorithm against inter-observer variability (IOV)
(5) correlation analysis between geometric and dosimetric evaluation measures to determine whether these are coherent.

To our knowledge, this study is the first to combine all of these components to investigate the use of auto-segmentation in an MR-guided RT scenario. By automatically generating treatment plans we can increase treatment plan comparability. The IOV measure provides a benchmark of our algorithm. Furthermore, this workflow can easily be adapted to evaluate any auto-segmentation approach within the scope of RT.

## 2. Materials & methods

**Figure 1.**
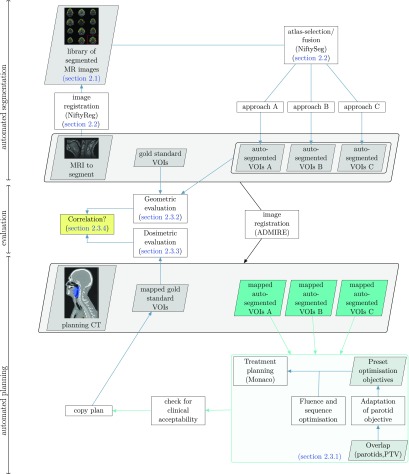
Illustration of the full workflow established in this work. The top part illustrates the auto-segmentation, the central part the geometric and dosimetric evaluations and the bottom part the automated planning. Each of these steps is performed following a leave-one-out cross-validation strategy. Related sections of this article are annotated.

Figure 1 provides an overview of the workflow established in this study with references to the respective sections that detail the individual steps. We first performed three different atlas-based segmentation methods using a library of manually segmented MR images, which is illustrated in the top part of figure 1. We then warped each set of auto-segmented VOIs into the geometric space of the corresponding CT using a deformable image registration (central part of figure 1). Afterwards, we automatically generated clinically acceptable treatment plans for each of these warped sets and copied the obtained treatment plans to the corresponding set of manually segmented VOIs (bottom part of figure 1). The central part of figure 1 shows the geometric and dosimetric evaluations, covered in sections 2.3.2 and 2.3.3, respectively, with the auto-generated segmentations and treatment plans as input. Finally, we investigated the correlations between geometric and dosimetric evaluation measures, as highlighted in the yellow box.

### 2.1. Data acquisition and preparation

**Figure 2.**
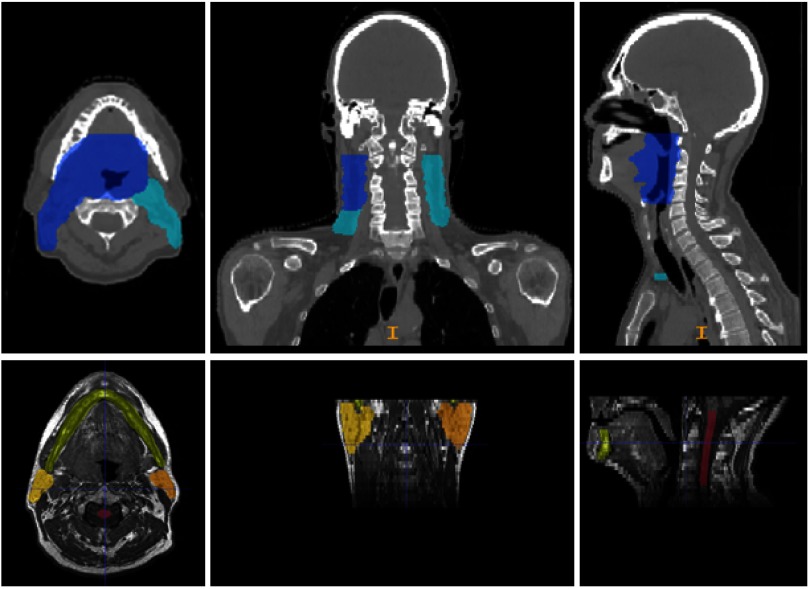
This figure depicts axial, coronal and sagittal slices of the CT (top row) and MR (bottom row) images of one example patient from the database used in this study. The coloured regions represent the manual segmentations of the primary planning target volume (PTV) (blue) and the secondary PTV (turquoise) on the CT, as well as the left (orange) and right (yellow) parotids, the mandible (green) and the spinal cord (red) on the MR images.

We used a retrospectively acquired library of 12 T1-weighted (T1w) pre-treatment MR images and same-day CT scans. All 12 patients had a tumour at the base of the tongue and were treated at the MD Anderson Cancer Center (Houston, Texas, USA). The respective image acquisition parameters are provided in table 1. A clinician manually delineated four VOIs on the T1w MR images: the left and the right parotid, the spinal cord and the mandible. Two additional clinicians manually delineated the primary (including involved lymph nodes) and secondary (including non-involved lymph nodes) clinical target volumes (CTVs), the optical nerves and lenses, the chiasm and the brainstem on the CT images. All VOIs were delineated using the treatment planning system (TPS) Raystation (Raysearch, Stockholm, Sweden). Figure 2 illustrates one example image set together with the manual segmentations.

**Table 1.** Imaging parameters of the MR and CT images used in this study.

Parameter	MR	CT
FOV (#pixels)	$512 \times 512$	$512 \times 512$
#slices	30	[165, 235]
Voxel size (mm^3^)	$0.5 \times 0.5 \times 4$	$0.98 \times 0.98 \times 2.5$
TE (ms)	[6.54, 7.85]	n.a.
TR (ms)	[601, 800]	n.a.
Flip angle (°)	90	n.a.
Sequence type	2D T1w spin echo	n.a.
Field strength/tube voltage	3 T	120 keV

### 2.2. Automated segmentation

We chose atlas-based auto-segmentation approaches making use of the software tools NiftyReg (Modat *et al* 2010, 2014) and NiftySeg (Van Leemput *et al* 1999, Cardoso *et al* 2011), both developed at the University College London (United Kingdom). The workflow referring to the auto-segmentation is illustrated in the top part of figure 1.

In the following, we define an *atlas* as a library of MR images, paired with previously segmented VOIs. We call the previously unseen MR image the *target image*. Atlas-based segmentation consists of two main steps: image registration of all library images to the target image and a subsequent fusion of individual segmentation results from each atlas to a common segmentation of the target image. We performed the registration in two steps: an affine initialisation with a block-matching algorithm (Modat *et al* 2014), followed by a deformable registration with a free-form deformation (FFD) algorithm (Modat *et al* 2010).

For the affine registration, the atlas and target image were each divided into blocks of $4\times4\times4$ voxels. In an iterative procedure, each block in the target image was compared to corresponding neighbouring blocks in the atlas image. For the most similar block in terms of its normalised cross correlation (NCC), the transformation parameters were determined using a least-trimmed square regression method with 12 degrees of freedom. The deformable registration used a fast FFD algorithm with B-splines. The atlas and target image were divided into control position points (CPPs) using a multi-resolution approach. The CPPs in the target image were optimised via an objective function that incorporated the image similarity through the NCC and a penalty term to ensure smoothness and avoid folding.

After the registration of all library images to the target image, we compared three atlas selection and fusion approaches to obtain the final segmentation. In all three approaches, we determined the similarity between two images by calculating the NCC coefficient.

In the best atlas approach (approach A) we selected the library image which was most similar to the target image. Approach B was a weighted majority voting method. For each voxel, the labels of the registered library images were combined into a single label with a weighted majority voting. The weights were derived locally from the similarity between library and target image (Cardoso *et al* 2015). Locally was defined as the application of a Gaussian kernel with a standard deviation (SD) of 2.5 voxels around each voxel. We call this the multi-atlas weighted majority voting (maWMV) approach. Approach C was the multi-atlas Similarity and Truth Estimation for Propagated Segmentations (maSTEPS) (Cardoso *et al* 2013) and is closely related to the well-established STAPLE method (Warfield *et al* 2004). STEPS consists of seven main steps:

(1) All library images are registered to the target image.
(2) For each voxel, the n library images which locally are most similar to the target image are chosen.
(3) An initial ground truth estimation of the segmentation is determined using a majority voting approach.
(4) The sensitivity and specificity with respect the initial segmentation in (3) are determined for the chosen atlases and a weight is assigned for each atlas accordingly.
(5) The ground truth estimation of the segmentation is updated with a weighted majority voting using the weights from (4).
(6) If all atlases agree on a label, this voxel is declared as solved and removed from the estimation.
(7) Steps (3)–(6) are repeated until convergence.

We chose n  =  5 for (2) as it had the optimal performance for the data used in this study.

We determined computation times for a programme execution on an Intel® Xeon® CPU E5-1660v3 (3 GHz) processor.

### 2.3. Planning study

To evaluate the geometric and dosimetric accuracy of the auto-segmentation methods, we devised a planning study based on a leave-one-out cross-validation strategy: We performed the three auto-segmentation methods for each patient of the library described in section 2.1, where the MR image of the respective patient was excluded from the library and used as the target, with the atlas library comprising the remaining MR images. The manually segmented VOIs (parotids, spinal cord and mandible) of one clinician served as the gold standard.

To investigate the impact of segmentation differences between auto-segmented and gold standard VOIs on planned dose volume parameters, we generated treatment plans for all auto-segmented VOIs and superimposed the dose distributions on the gold standard VOIs. Due to the restricted coverage in the superior-inferior direction and the lack of electron density information of the MR images, we warped the automatically and manually segmented OARs from the MR images to the corresponding CT scans by using the deformable registration framework ADMIRE (research version 1.1, Elekta AB, Stockholm, Sweden). Furthermore, we included the brainstem, the optical nerves and lenses, the chiasm, as well as the CTVs in the treatment planning. We expanded the CTVs with a margin of 3 mm to obtain the PTVs. The brainstem and the spinal cord were expanded with a margin of 3 mm, the optical nerves and chiasm with a margin of 1 mm for the planning risk volumes.

#### 2.3.1. Automated treatment planning

To increase treatment plan comparability we implemented an automated plan generation approach making use of the research scripting interface of the TPS Monaco (research version 5.19.03, Elekta AB, Stockholm, Sweden, (Clements *et al* 2018)). The auto-planning approach is illustrated in the turquoise box in figure 1. With this approach we generated treatment plans for a 9-beam step and shoot IMRT treatment on the Unity MR-Linac (Elekta AB, Stockholm, Sweden) prescribing mean doses of 65 Gy to the primary PTV and 54 Gy to the secondary PTV in 30 fractions, following the INSIGHT study protocol (Welsh *et al* 2015). Details on the clinical goals are listed in the appendix in table A1. To calculate dose we used the GPU-based Monte Carlo dose engine (research version of GPUMCD, Elekta AB, Stockholm, Sweden, (Hissoiny *et al* 2011)) and chose the MR-Linac beam model for a magnetic field of 1.5 T. We normalised each dose distribution so that 95% of the primary PTV was covered by 95% of the prescribed dose.

We defined a template cost function that incorporated optimisation objectives on the target volumes and OARs. As the sparing of the parotids was difficult to achieve for our set of patients due to the large overlap with the target volumes, we chose to loosen the optimisation objective, as well as the clinical goal for the parotids. We determined the objective as a function of the overlap volume OV with the primary PTV:

1 \begin{align*} \newcommand{\e}{{\rm e}} \displaystyle D_{\rm mean}({{\rm OV~(\%)}})~\mathop{&lt;}\limits^{!}~24~({\rm Gy}) + 0.6~({\rm Gy})\cdot{{\rm OV}}~(\%). \nonumber \end{align*}

This approach has proven to be useful in clinical practice as suggested by Hunt *et al* (2006). It emulates the clinical reality at our hospital, where target coverage and the sparing of the brainstem, the spinal cord, as well as the optical structures are prioritised over a reduction of dose to the parotids.

The dose distribution, obtained through fluence and sequence optimisations in Monaco (research version 5.19.03, Elekta AB, Stockholm, Sweden), was then checked for clinical acceptability. We implemented an automated plan check algorithm to analyse whether all imposed clinical goals were fulfilled, using the research interface in Monaco (research version 5.19.03, Elekta AB, Stockholm, Sweden). Additionally, a clinician visually inspected the dose distributions.

The evaluation workflow is illustrated in the central part of figure 1, with inputs from the top and bottom part.

#### 2.3.2. Geometric evaluation

As a first indication of agreement we calculated the volume of each auto-segmented VOI, averaged over all patients and compared to the volume of the gold standard VOIs. Furthermore, we calculated four well-established geometric measures between the auto-segmented and the gold standard VOIs: the Dice similarity coefficient (DSC) (Dice 1945) for volumetric differences, and the standard (HD) and 95th percentile of the Hausdorff distance (HD95), and the mean surface distance (MSD) (Pekar *et al* 2010) for distance related differences. The DSC ranges from 0 to 1, where 1 indicates perfect overlap. The lower the HD, HD95 and MSD, the better the agreement.

#### 2.3.3. Dosimetric evaluation

To determine the dosimetric impact of segmentation differences between manually and automatically segmented VOIs, we calculated dose differences between dose volume parameters, where we normalised to the respective clinical goal $D_{\rm x, goal}$:

2 \begin{align*} \newcommand{\e}{{\rm e}} \displaystyle \Delta D_{\rm x,norm}=\frac{D_{\rm x,auto}({\rm Gy})-D_{\rm x,manual}({\rm Gy})}{D_{\rm x,goal}({\rm Gy})}. \label{eq:dosdiff} \nonumber \end{align*}

Index x denotes the type of dose volume parameter, e.g. the maximum dose to a certain fraction of the volume or the mean dose. For the parotids we calculated the difference between mean doses, where we normalised to a non-adapted clinical goal of 26 Gy. The spinal cord and the mandible were evaluated in terms of the maximum dose to 1 cm^3^ volume with clinical goals of 46 and 67.25 Gy, respectively. A negative $\Delta D_{\rm x, norm}$ means that a larger dose would be delivered to the gold standard compared to what was planned for the auto-segmented VOIs.

#### 2.3.4. Geometric measures as predictors for dosimetric accuracy

To determine whether geometric measures, can reliably predict the dosimetric impact on planned dose volume parameters, we investigated the correlation between the geometric and dosimetric quantities by calculating Spearman’s correlation coefficients (Spearman 1904). We calculated the correlation coefficients individually for the three different auto-segmentation approaches as these were determined for the same set of patients and could therefore not be treated as independent. Additionally, we performed a qualitative analysis by visual inspection of individual patient images in order to understand the dependency of the correlation on the shape and the size of the OAR, the dose metric, as well as the relative position to the target volume (i.e. location within large dose gradients).

### 2.4. Inter-observer variability (IOV)

It is a known problem that the evaluation of auto-segmentation suffers from the lack of an objective ground truth. IOV can provide an estimate of the upper bound on the desired auto-segmentation accuracy. To determine this for the data used in this study, two additional observers contoured all VOIs on all patient images. Each of the observers followed the contouring guidelines defined in Sun *et al* (2014). We estimated the IOV geometrically and dosimetrically. To determine the geometric IOV between two observers we first calculated the DSC, HD, HD95 and MSD between the respective observers’ contours for each patient and defined the IOV as the average and SD over all patients. The overall IOV was then calculated as the average of the three individual IOVs, with the SD being the root mean square (RMS) of the three individual SDs. To determine the dosimetric IOV, we chose approach B as a representative approach for the auto-segmentation. We superimposed the respective dose distribution on each of the three sets of manually segmented VOIs. For each patient and VOI, we approximated the dosimetric variability with the SD of the three ‘manual’ dose values, normalised to the clinical goal. We estimated the overall variability by calculating the mean and SD over all patients.

### 2.5. Statistical evaluation

Tests for statistically significant differences were performed using Student’s paired t-test (Student 1908) at a significance level of p  =  0.05/3 with a Bonferroni correction to account for multiple comparisons. Since a condition of the paired t-test is the normal distribution of the data, we tested the results for normality by visual inspection of Q-Q-plots. All analyses were performed within an in-house developed Python software.

## 3. Results

The computation of the full auto-segmentation process took less than an hour. A major part was attributed to the image registration. The image registration between two images took 5 min on average. This resulted in a total time of 55 min for our library of 11 patient images for the registration part. The only difference between approach A (best atlas) and the approaches B and C (maMWV and maSTEPS) in terms of the computation time was attributed to the atlas selection and fusion method. Selecting the most similar atlas in approach A did not add any significant time. The atlas fusion for approaches B and C added less than a minute for the full database.

**Figure 3.**
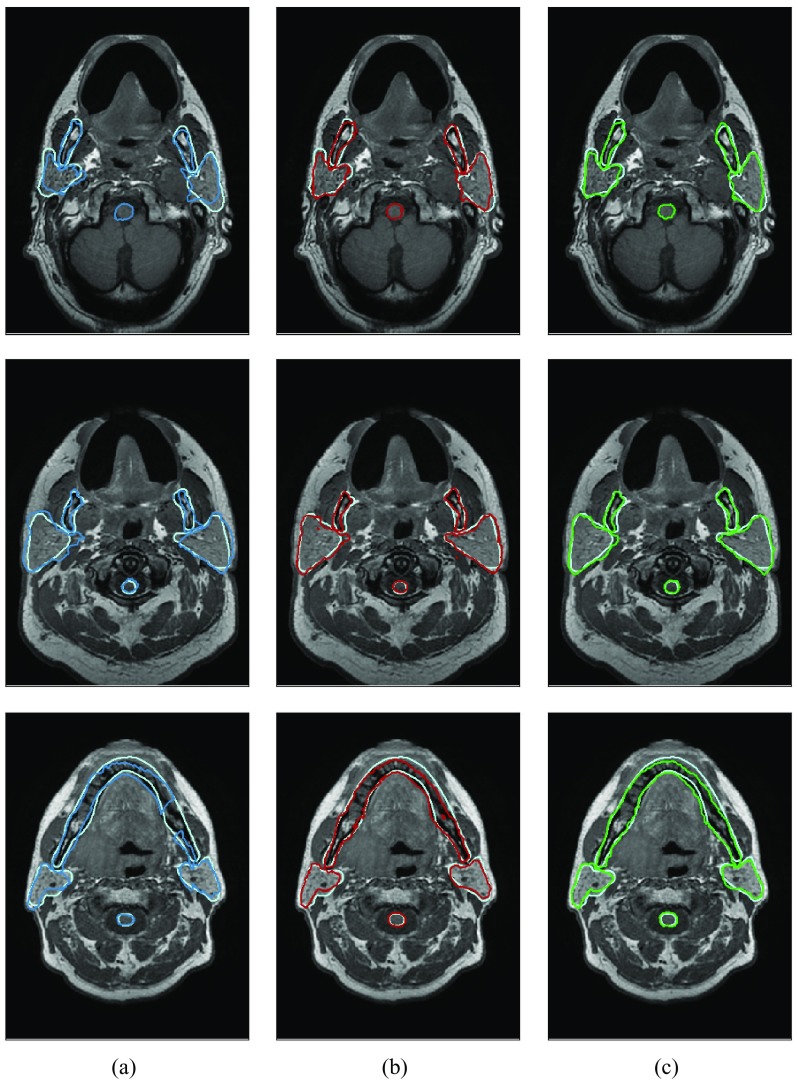
This figure shows in each row a typical example comparing the manual segmentation (light blue) to (a) approach A (dark blue), (b) approach B (red) and (c) approach C (green), respectively. Each example originates from a different patient image.

Figure 3 provides three typical examples from three different patients for a qualitative comparison of all three auto-segmentation approaches to the gold standard. The two multi-atlas approaches (columns 2 and 3) clearly outperformed the best-atlas approach (first column) in all shown cases.

### 3.1. Geometric evaluation

Table 2 lists the mean volume, as well the SD for all VOIs and segmentation approaches. The intervals of mean values  ±1 SD of manually and auto-segmented volumes overlapped for all cases.

**Table 2.** Automatically segmented mean volumes with standard deviations for all approaches and volumes of interest (VOI) with comparisons to manually segmented (gold standard) volumes.

VOI	Manually segmented volume (cm^3^)	Approach	Auto-segmented volume (cm^3^)
Right parotid	29.11 ± 8.89	A (best atlas)	31.29 ± 12.07
		B (maWMV)	29.03 ± 8.24
		C (maSTEPS)	29.62 ± 7.70

Left parotid	27.58 ± 5.22	A (best atlas)	30.92 ± 9.43
		B (maWMV)	29.75 ± 6.98
		C (maSTEPS)	30.67 ± 7.07

Spinal cord	6.34 ± 1.45	A (best atlas)	6.54 ± 1.32
		B (maWMV)	5.94 ± 0.92
		C (maSTEPS)	6.76 ± 1.11

Mandible	66.93 ± 18.53	A (best atlas)	54.74 ± 13.71
		B (maWMV)	60.92 ± 16.87
		C (maSTEPS)	61.86 ± 16.77

**Figure 4.**
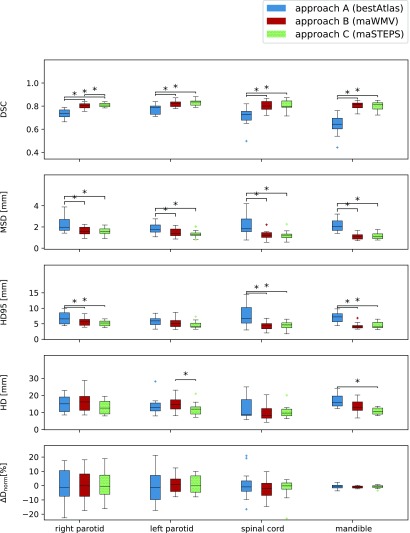
Boxplots of, from top to bottom, the DSC, HD95, HD and dosimetric difference $\Delta D_{\rm norm}$ for all OAR (*x*-axis) and automated segmentation approaches (A in blue, B in red and C in green). The boxes indicate the interquartile range (IQR), the whiskers extend to the minimum and maximum values. Outliers are defined as data points beyond 1.5 IQRs from the IQR, denoted with a plus sign. Stars indicate statistical significance (p  <  0.05/3).

The top four rows of figure 4 illustrate boxplots of the DSC, HD, HD95 and MSD for all VOIs and the three atlas fusion methods. The stars indicate statistical significance. Table 3 lists the mean and standard deviations for all applied geometric measures. The IOV was included as a reference value.

**Table 3.** Geometric evaluation for all VOIs and auto-segmentation approaches: mean values for DSC, HD, HD95 and MSD. All mean values have been calculated by averaging over all 12 patients. For a reference, we also include the inter-observer variability (IOV), derived from the manual contours of three different observers.

VOI	Approach	$\overline{DSC}$	$\overline{HD}$ (mm)	$\overline{HD95}$ (mm)	$\overline{MSD}$ (mm)
Right parotid	A	0.74 ± 0.04	15.07 ± 5.03	6.84 ± 1.95	2.24 ± 0.75
	B	0.80 ± 0.03	16.51 ± 6.96	5.65 ± 1.41	1.61 ± 0.43
	C	0.81 ± 0.02	13.33 ± 5.20	5.20 ± 0.97	1.56 ± 0.38
	IOV	0.84 ± 0.04	10.76 ± 4.35	4.97 ± 1.66	1.40 ± 0.45

Left parotid	A	0.77 ± 0.04	13.89 ± 5.36	5.84 ± 1.64	1.84 ± 0.54
	B	0.82 ± 0.03	15.00 ± 4.62	5.17 ± 1.62	1.47 ± 0.41
	C	0.83 ± 0.03	12.13 ± 3.91	4.63 ± 1.21	1.35 ± 0.40
	IOV	0.83 ± 0.04	10.94 ± 3.75	5.27 ± 1.76	1.59 ± 0.63

Spinal cord	A	0.71 ± 0.08	12.72 ± 3.91	7.68 ± 3.56	2.26 ± 1.10
	B	0.80 ± 0.05	10.12 ± 4.83	4.26 ± 1.36	1.24 ± 0.45
	C	0.80 ± 0.05	10.35 ± 3.75	4.39 ± 1.33	1.21 ± 0.44
	IOV	0.79 ± 0.07	7.12 ± 5.15	4.64 ± 3.06	1.55 ± 0.81

Mandible	A	0.64 ± 0.09	16.65 ± 3.60	6.96 ± 1.84	2.14 ± 0.60
	B	0.80 ± 0.04	13.33 ± 4.06	4.31 ± 1.05	1.10 ± 0.28
	C	0.80 ± 0.04	10.88 ± 2.07	4.44 ± 1.09	1.35 ± 0.30
	IOV	0.85 ± 0.04	8.94 ± 3.16	3.85 ± 1.56	0.92 ± 0.45

The mean DSC for approach A ranged from 0.64 to 0.77. We found statistically significant improvements when using the multi-atlas approaches B and C with a mean DSC larger than 0.80 for all VOIs. Differences between the mean DSC values ranged from 0.05 for the parotids to 0.16 for the mandible. This superior performance of the multi-atlas approaches also held true for the mean MSD, ranging from 1.10 mm to 1.61 mm compared to 1.84 mm to 2.26 mm, and the mean HD95, ranging from 5.84 to 7.68 mm (approach A) compared to 4.26 to 5.65 mm (approaches B and C). The mean HD ranged from 10.88 to 16.65 mm for all approaches. The only significant differences in the HD could be detected between approaches B and C for the left parotid and between A and B for the mandible. We found a trend towards smaller SDs for all quantitative measures and VOIs when applying multi-atlas approaches. When using the multi-atlas approaches (B and C), the mean values of all geometric measures for the parotids and the spinal cord were within one SD of the IOV. The auto-segmentation performance for the mandible was slightly worse than the IOV. The best-atlas approach (A) had a lower accuracy than the IOV.

### 3.2. Dosimetric evaluation

The bottom row of figure 4 shows the dosimetric differences, calculated using equation (2). Table 4 lists mean and SDs, averaged over all patients. Furthermore, we included the dosimetric variability, calculated as described in section 2.4. Overall, no method was superior to any other in terms of dosimetric differences. Dose differences took both positive and negative values but were close to a zero mean for all VOIs and segmentation approaches. Differences as large as 23% of the clinical goal in either direction were observed for the parotids. Dose differences to the mandible were below 4% of the clinical goal. The SDs of the dosimetric differences were within the range of the dosimetric variability, which means that the overall dosimetric accuracy was comparable to the IOV. However, in half of the patients for the parotids and the spinal cord, and in 75% for the mandible the individual dosimetric difference was outside the range of the dosimetric variability.

**Table 4.** Normalised dosimetric differences $\Delta D_{\rm norm}$ (equation (2)), as well as dosimetric variability (section 2.4). A negative $\Delta D_{\rm norm}$ means a larger mean dose to gold standard structures. For a reference, we also include the inter-observer variability (IOV).

VOI	Approach	$\overline{\Delta D_{\rm norm}}$ (%)	IOV (%)
Right parotid	A	0.06 ± 12.93	
	B	−0.84 ± 10.82	5.56 ± 4.78
	C	0.02 ± 10.26	

Left parotid	A	−0.65 ± 11.39	
	B	0.83 ± 6.51	6.00 ± 3.93
	C	0.68 ± 6.28	

Spinal cord	A	0.95 ± 10.68	
	B	−2.77 ± 6.64	4.76 ± 4.58
	C	−2.17 ± 7.41	

Mandible	A	−0.66 ± 1.64	
	B	−1.02 ± 0.85	0.46 ± 0.26
	C	−0.84 ± 1.18	

### 3.3. Geometric measures as predictors for dosimetric accuracy

**Figure 5.**
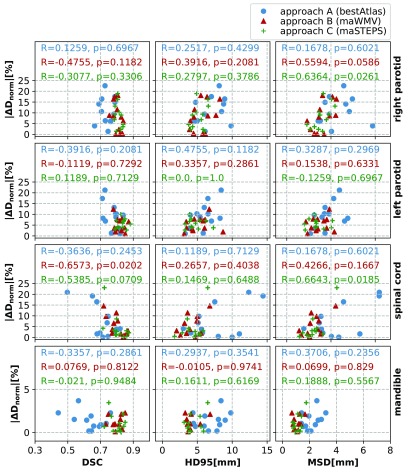
Scatter plots illustrating dose differences between manually and auto-segmented VOIs normalised to the clinical goal as a function of the respective geometric measures (from left to right: DSC, HD95 and MSD), separated according to the VOIs used in this study (from top to bottom: right parotid, left parotid, spinal cord and mandible). The different colours and symbols illustrate the three auto-segmentation methods of this study. The numbers in each subplot are the respective correlation coefficients R together with the p-values, calculated using Spearman’s approach.

Figure 5 depicts the absolute values of the dosimetric differences as a function of three geometric measures (DSC, MSD, HD95) for all VOIs and segmentation approaches. For a qualitative overall picture, we illustrate all approaches in the same subfigures. The correlation coefficients for each approach are included in each subfigure.

If geometric measures were good predictors for the impact of segmentation inaccuracies on the dose distribution, we would expect large negative correlation coefficients R for the DSC and large positive R for distance-related measures. However, for the dataset here, correlations were small with R^2^  <  0.5 and did not have the expected sign in all cases, e.g. a negative correlation existed between the MSD and $|\Delta D|$ for the left parotid, segmented using approach C.

As the HD is very sensitive to outliers we only included the HD95 in figure 5. We obtained even smaller correlation coefficients when analysing the dosimetric differences as a function of the HD (data not shown here).

**Figure 6.**
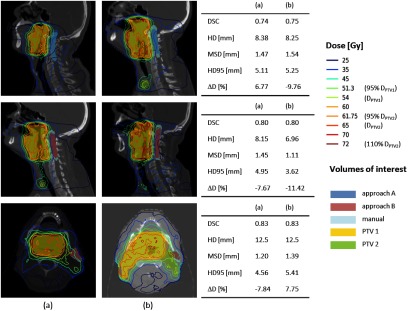
This figures illustrates three example cases where the geometric differences (DSC, HD, HD95 and MSD) were similar between the patients in columns (a) and (b) but the dosimetric impact differed, as shown in the tables in the last column. The first two rows illustrate examples for the spinal cord, the last row for the left parotid. Coloured lines represent selected isodose lines with the prescribed doses to the target volumes indicated in brackets. Coloured areas illustrate relevant VOIs: auto-segmented OARs using approach A (blue) and approach B (red), respectively, manually segmented OARs (light blue), primary PTV (yellow) and secondary PTV (green).

With the qualitative per-patient analysis we found that larger dosimetric differences started to appear with the OAR being closer to the target volume. However, there was only a small and non-significant correlation when clustering the data as a function of the distance to the target volume (data not shown here). Figure 6 illustrates three example pairs of cases with similar geometric accuracy yet large deviations between the dosimetric differences. The first two columns show a sagittal or axial image plane for two different patients. The coloured lines represent the isodose curves, whereas the coloured areas show the manually and automatically segmented VOIs. The respective geometric and dosimetric differences between manually and automatically segmented VOIs are provided in the table in the third column. The first two rows illustrate examples for the spinal cord, where steep dose gradients have a large influence due to the nature of the clinical goal (maximum dose). The last row shows an example for the parotid, where the relative position to the high dose region largely impacts the dosimetric outcome.

## 4. Discussion

### 4.1. Geometric evaluation

Both multi-atlas approaches outperformed the best-atlas approach in terms of the geometric accuracy (DSC, HD95 and MSD). This finding is in line with other published studies (Han *et al* 2008, Teguh *et al* 2011, Daisne and Blumhofer 2013). Comparing the two multi-atlas approaches B and C, there was no clear benefit of using one or the other. As these two approaches only differ in the atlas fusion method, we can conclude that for the data utilised in this study, the performance of atlas-based approaches was mainly influenced by the quality of the image registration and choosing a local instead of a global approach (atlas fusion in the multi-atlas approaches versus global atlas selection in approach A). The HD was not a reliable measure for the geometric accuracy of the data used in this study. As this measure provides the maximum distance to the gold standard segmentations, it is very sensitive to outliers and is hence not a good representative of the overall geometric accuracy.

To compare our results with published auto-segmentation studies, table 5 lists mean reported geometric measures. The majority of the reported studies used CT scans. Only three studies chose MR imaging as their imaging modality (Yang *et al* 2014, Veeraraghavan *et al* 2015, Wardman *et al* 2016). As none of these studies calculated the HD95, we did not include this measure in table 5.

**Table 5.** This table lists geometric measures (mean DSC, mean Hausdorff distance (HD) and mean surface distance (MSD) reported for the volumes of interest (VOI) of this work. The mean values for the parotids are averaged between the left and right parotid.

VOI	DSC	HD (mm)	MSD (mm)	modality	#patients	Study
Parotids	0.76	14.48	2.04	MR	12	This study (A)
	0.81	15.75	1.54	MR	12	This study (B)
	0.82	12.73	1.46	MR	12	This study (C)
	0.79	—	4.97	MR	14	Wardman *et al* (2016)
	0.77	—	—	CT	10	Beasley *et al* (2016)
	0.65	45	—	CT	100	Hoang Duc *et al* (2015)
	0.84	13	—	CT	18	Fritscher *et al* (2014)
	0.91	3.46	0.31	MR	15	Yang *et al* (2014)
	0.72	15	2.5	CT	20	Daisne and Blumhofer (2013)
	0.79	—	—	CT	5	La Macchia *et al* (2012)
	0.79	—	2.5	CT	10	Teguh *et al* (2011)
	0.83	5.8	—	CT	25	Qazi *et al* (2011)
	0.86	4.95	—	CT	25	Pekar *et al* (2010)
	0.68	—	—	CT	13	Sims *et al* (2009)
	0.85	—	—	CT	10	Han *et al* (2008)

Spinal cord	0.71	12.72	2.26	MR	12	This study (A)
	0.80	10.12	1.10	MR	12	This study (B)
	0.80	10.35	1.35	MR	12	This study (C)
	0.37	—	17.5	MR	14	Wardman *et al* (2016)
	0.75	40	—	CT	100	Hoang Duc *et al* (2015)
	0.81	—	—	CT	5	La Macchia *et al* (2012)
	0.78	—	2.3	CT	10	Teguh *et al* (2011)
	0.75	—	—	CT	10	Han *et al* (2008)

Mandible	0.64	16.65	2.14	MR	12	This study (A)
	0.80	13.33	1.10	MR	12	This study (B)
	0.80	10.88	1.35	MR	12	This study (C)
	0.86	—	—	CT	5	La Macchia *et al* (2012)
	0.93	—	2.64	CT	25	Qazi *et al* (2011)
	0.78	—	—	CT	13	Sims *et al* (2009)
	0.9	—	—	CT	10	Han *et al* (2008)

With a mean DSC larger than 0.8 and a mean MSD smaller than 2 mm, our multi-atlas methods lie in the range of reported values, as well as within one SD of the IOV that has been determined for the data in this study. Published results for the HD are sparse and have large variations. Our study is the only one reporting on the HD for the mandible. For the parotids, our results are comparable to Daisne and Blumhofer (2013) and Fritscher *et al* (2014). For the spinal cord, we found a lower HD than Hoang Duc *et al* (2015).

The segmentation accuracy in terms of the DSC of the mandible was slightly worse in our approach compared to reported studies (Han *et al* 2008, Qazi *et al* 2011, La Macchia *et al* 2012). This may be attributed to the fact that each of these studies was conducted using CT images. As the mandible is a bony structure, it is more clearly visualised on CT images.

The results published by Yang *et al* (2014) demonstrate a superior performance of their algorithm. They used an atlas-based approach, refined by a machine learning post-processing step. However, in contrast to our study, they applied their approach to the auto-segmentation of post-RT MRIs using pre-RT MRIs from the same patient. This results in a smaller expected variance between atlas and target images.

### 4.2. Dosimetric evaluation

None of the three auto-segmentation approaches chosen in this work was superior to any other in terms of dosimetric accuracy for any of the investigated OARs. Average absolute dose differences were below 3% of the clinical goal for all OARs and segmentation approaches. However, dose differences for different patients were widely spread with a SD of up to 11% of the clinical goal. Despite these large SDs, we found that the dosimetric accuracy was comparable to the dosimetric IOV.

Several groups have addressed the need for quantifying the impact of inaccurate localisations of VOIs on the planned dose distribution when using auto-generated contours for the treatment plan and creation process. These can be summarised into essentially three approaches.

The first approach is to use existing planned dose distributions on gold standard VOIs and superimpose these on the auto-segmented VOIs. The effect of delineation variations on dose parameters can then be determined by comparing dose differences to paired gold standard and auto-segmented VOIs. This method was applied by Eldesoky *et al* (2017) for the segmentation of breast tissues and by Conson *et al* (2014) for the segmentation of brain structures. A limitation of applying this method to the plan creation is that instead of generating new treatment plans for the automatically segmented VOIs, the original plans are used, therefore ignoring the fact that different contours imply a different optimisation problem.

The second approach individually optimises the dose distributions for both auto-segmented and gold standard VOIs, using the same beam parameters and planning constraints. Tsuji *et al* (2010) applied this approach for pairs of pre- and mid-treatment CTs of the H&N region. A limitation of this method is that instead of comparing the direct dosimetric impact of segmentation inaccuracies, two separately generated treatment plans are compared.

The third approach is to create treatment plans for the auto-segmented sets of VOIs and superimpose the dose distributions on the gold standard VOIs. Nelms *et al* (2012) applied this approach to investigate effects of inter-observer variabilities in manual OAR segmentations from 32 observers. A drawback of their study is that they only use the CT image of one patient for their evaluation. Voet *et al* (2011) applied the third approach to investigate whether geometric measures can predict the amount of underdosage in the PTV. Auto-segmented H&N VOIs edited by clinicians served as the gold standard. They included the neck levels and the parotids in their analysis. Beasley *et al* (2016) compared dosimetric differences and the geometric accuracy of auto-generated contours for the parotids and the larynx of 10 H&N cancer patients, using the manually drawn contours of 5 observers as gold standard.

In this study, we chose the third approach. We found that this was the only appropriate approach to use as it solves the optimisation problem directly for the auto-segmented VOIs. This emulates the clinical reality in the case of an application to treatment plan generation.

In contrast to our findings, Voet *et al* (2011) reported on a small, statistically non-significant dose difference for the parotids (−0.8  ±  1.1 Gy, i.e. SD  <  3%). With respect to the target volume (CTV) they found that the mean reduction in dose to 99% of the volume (D_99_) was large with 14.2 Gy (ranging from 1 to 54 Gy). Beasley *et al* (2016) reported on an average difference in the mean dose to the parotids between auto-generated and gold standard VOIs, relative to the latter, of  −4.8  ±  3.4% ranging from $-18\%$ to 43%. They also compared mean doses for the larynx and found a difference of  −8.4  ±  2.3%, ranging from $-20\%$ to 3%. The uncertainty was determined by the IOV between 5 observers. These large ranges of dosimetric differences are in line with our findings. Tsuji *et al* (2010) did not find any significant dose differences to the manually and automatically segmented OARs. However, they used the second approach, therefore impairing a direct comparison.

### 4.3. Geometric measures as predictors for dosimetric accuracy

In order to understand whether the geometric measures used in our study (DSC, HD, HD95 and MSD) can be a reliable surrogate for dosimetric differences and treatment planning accuracy, we investigated their correlation. Voet *et al* (2011) showed that both DSC and mean contour distances did not have a large predictive value with respect to their influence on dose coverage of the target volume. They reported that an underdosage of 11 Gy may appear even for a decent geometric accuracy with DSC  =  0.8 and ASD  <  1 mm. Eldesoky *et al* (2017) investigated the correlation between geometric and dosimetric accuracy for four target volumes in breast cancer RT. They found a small significant correlation for only one of those target volumes between the DSC and dose volume metrics.

In contrast to the aforementioned studies, we were focusing on OARs instead of target volumes. The results presented in figure 5, did not imply a strong correlation between these measures. This finding was also reflected in the small correlation coefficients. All patients in our study had a tumour at the base of the tongue. For this reason, relative positions of OARs and target volumes were similar. Despite this similarity, the relation between dose deposition and the location of target volumes remained to be very complex. The visual inspection of individual patient images suggests that the impact of geometric inaccuracies on dosimetric outcome is influenced by the shape of the structure, the type of clinical goal (maximum or mean dose) and the location of geometric differences (i.e. whether these lie within regions of high dose gradients or are far from those). Examples of high dose gradients influencing the correlation between geometric and dosimetric measures could be seen in the first two example cases in figure 6.

These findings suggest that for the data used in this study the investigated geometric measures are not reliable surrogates for the dosimetric outcome. The correlation values for the DSC are in line with results reported by Beasley *et al* (2016). Additionally, they found a large correlation (R  =  0.83) between the centroid distance and the differences in the mean dose to the parotids. However, evaluating this for the data in this study, we did not find such a strong correlation. Furthermore, correlations with the distance-related measures were smaller compared to Beasley *et al* (2016).

While the SD of dosimetric differences for the full patient cohort was within the range of the dosimetric IOV, we found that for individual patients, the dosimetric difference was outside this variability despite a decent geometric accuracy. This finding highlights the need to carefully investigate the dosimetric impact of segmentation inaccuracies.

### 4.4. Limitations and future work

One limitation of this study is the relatively small number of available training data. Considering the large appearance variations between different patients’ anatomies, a larger database would be needed to account for these variations. However, a larger database would not invalidate the conclusions on the accuracy of the atlas-based segmentations. Instead, we would expect a higher geometrical accuracy, as more variation in the library will also more likely include images similar to the target image.

Furthermore, due to the small imaging coverage of the patients’ anatomies in the superior-inferior direction we could only include four OARs in our analysis. Treatment planning of H&N requires the segmentation of more OARs such as the optical structures and the brainstem.

It is a known problem that the evaluation of auto-segmentation suffers from the lack of an objective ground truth. While we determined the IOV to provide an estimate of the upper bound on the desired auto-segmentation accuracy, we chose the contours of one observer as the gold standard VOIs to compare to. This observer was the clinician whose contours were used to create the atlas for the auto-segmentation. Previous publications suggested to combine the contours of several observers into one common contour, for example by using an approach called Simultaneous Truth and Performance Level Estimation (STAPLE) (Warfield *et al* 2004). With STAPLE one could obtain a gold standard that might be closer to the unknown ground truth by considering the agreement between different observers on the absence or presence of the VOI at a certain location within the image. In future work one could consider using the STAPLE of several observers as the gold standard VOIs, both, as input for the atlas-based segmentation, as well as a reference to compare to.

A limitation of the atlas-based segmentation approach is the computation time. With computation times of an hour using a library of 11 images this would not be suitable for an online workflow. However, the use of a multi-atlas approach for the offline segmentation of pre-treatment images would already represent a significant time-gain compared to manual segmentations which can take up to several hours. In an adaptive RT workflow, one could then use previous, already segmented, images of the same patient in a single-atlas approach which would necessitate the registration of only one image to the target image and reduce time significantly to a few minutes. We furthermore expect that we can significantly reduce the registration time by changes in the algorithm itself, e.g. by parallelising image registrations for different library images and cutting down the time for the affine registration.

Dose calculations in this study were performed simulating a 9-beam step and shoot IMRT treatment on an MR-linac in a magnetic field. While other radiation delivery techniques may lead to slightly different dosimetric results, the dosimetric evaluation method is independent of the treatment type and can be easily applied to more patient data. The template approach established in this study worked well for all included patients. We anticipate some necessary changes of the template for very different anatomies compared to the patient data in this study.

In future work we would like to investigate new measures than can more reliably predict the dosimetric effect of segmentation inaccuracies. Anticipating the dosimetric effect from the geometric evaluation directly would remove the need to optimise treatment plans for each set of auto-segmented VOIs. On the other hand, using geometric measures that do not reliably predict the impact on the dose distribution limits their applicability in RT. One could incorporate knowledge about the position of OARs relative to target volumes to account for regions with sharp dose gradients. Furthermore, first applications of machine learning approaches in RT seem promising and could be applied for this problem by, for example, modelling geometric uncertainties using neural networks and determining the effect on dose distributions.

## 5. Conclusion

To our knowledge, this was the first study to investigate the use of contours derived from atlas-based segmentation on H&N MR images in the context of treatment plan generation for RT with a complete analysis of the geometric and dosimetric accuracy. We benchmarked the accuracy of the generated contours by determining the IOV for the image data used in this study. A geometric accuracy in the range of the IOV could be achieved, as well as clinically acceptable treatment plans. Multi-atlas approaches outperformed a simple best-atlas approach. Although there appeared to be a slight correlation between geometric (DSC, MSD and HD95) and dosimetric measures, the geometric measures alone were not sufficient to predict the dosimetric impact of segmentation inaccuracies on RT treatment plans.

## Appendix. Clinical treatment planning goals

**Table A1.** Clinical treatment planning goals according to our institution’s clinical protocol, prescribing a mean dose of 65 and 54 Gy to the primary and secondary planning target volumes (PTV), respectively. Depending on the clinical case, priorities 2a and 2b can change order.

Priority	VOI	Clinical goal
1	Spinal cord	D_1cc_ < 46 Gy
1	Spinal cord + 3 mm PRV	D_1cc_ < 48 Gy
1	Brainstem	D_1cc_ < 54 Gy
1	Brainstem + 3 mm PRV	D_1cc_ < 56 Gy
2a	Primary PTV	D_99%_ > 90% of 65 Gy
2a	Primary PTV	D_95%_ > 95% of 65 Gy
2a	Primary PTV	D_50%_ = 65 ± 1 Gy
2a	Secondary PTV	D_99%_ > 90% of 54 Gy
2a	Secondary PTV	D_95%_ > 95% of 54 Gy
2a	Secondary PTV	D_50%_ = 54 ± 1 Gy
2b	Optical nerves	D_1cc_ < 54 Gy
2b	Optical nerves + 1 mm PRV	D_1cc_ < 55 Gy
2b	Chiasm	D_1cc_ < 55 Gy
2b	Chiasm + 1 mm PRV	D_1cc_ < 56 Gy
2b	Optical lenses	D_mean_ < 6 Gy
3	Parotids	D_mean_ < 26 Gy

## References

[pmbaacb65bib001] Beasley W J, McWilliam A, Aitkenhead A, Mackay R I, Rowbottom C G (2016). The suitability of common metrics for assessing parotid and
larynx autosegmentation accuracy. J. Appl. Clin. Med. Phys..

[pmbaacb65bib002] Cardoso M J, Clarkson M J, Ridgway G R, Modat M, Fox N, Ourselin S (2011). LoAd: a locally adaptive cortical segmentation
algorithm. NeuroImage.

[pmbaacb65bib003] Cardoso M J, Leung K, Modat M, Keihaninejad S, Cash D, Barnes J, Fox N C, Ourselin S (2013). STEPS: Similarity and Truth Estimation for Propagated
Segmentations and its application to hippocampal segmentation and brain
parcelation. Med. Image Anal..

[pmbaacb65bib004] Cardoso M J, Modat M, Wolz R, Melbourne A, Cash D, Rueckert D, Ourselin S (2015). Geodesic information flows: spatially-variant graphs and their
application to segmentation and fusion. IEEE Trans. Med. Imaging 1.

[pmbaacb65bib005] Chung N N, Ting L L, Hsu W C, Lui L T, Wang P M (2004). Impact of magnetic resonance imaging versus CT on nasopharyngeal
carcinoma: primary tumor target delineation for radiotherapy. Head Neck.

[pmbaacb65bib006] Clements M, Schupp N, Tattersall M, Brown A, Larson R (2018). Monaco treatment planning system tools and optimization
processes. Med. Dosim..

[pmbaacb65bib007] Conson M, Cella L, Pacelli R, Comerci M, Liuzzi R, Salvatore M, Quarantelli M (2014). Automated delineation of brain structures in patients undergoing
radiotherapy for primary brain tumors: from atlas to dose-volume
histograms. Radiother. Oncol..

[pmbaacb65bib008] Daisne J F, Blumhofer A (2013). Atlas-based automatic segmentation of head and neck organs at
risk and nodal target volumes: a clinical validation. Radiat. Oncol..

[pmbaacb65bib009] Dice L R (1945). Measures of the amount of ecologic association between
species. Ecology.

[pmbaacb65bib010] Dirix P, Haustermans K, Vandecaveye V (2014). The value of magnetic resonance imaging for radiotherapy
planning. Seminars in Radiat. Oncol..

[pmbaacb65bib011] Edmund J M, Nyholm T (2017). A review of substitute CT generation for MRI-only radiation
therapy. Radiat. Oncol..

[pmbaacb65bib012] Eldesoky A R (2017). Dosimetric assessment of an Atlas based automated segmentation
for loco-regional radiation therapy of early breast cancer in the Skagen
Trial 1: a multi-institutional study. Clin. Transl. Radiat. Oncol..

[pmbaacb65bib013] Emami B, Sethi A, Petruzzelli G J (2003). Influence of MRI on target volume delineation and IMRT planning
in nasopharyngeal carcinoma. Int. J. Radiat. Oncol. Biol. Phys..

[pmbaacb65bib014] Faggiano E, Fiorino C, Scalco E, Broggi S, Cattaneo M, Maggiulli E, Dell’Oca I, Di Muzio N, Calandrino R, Rizzo G (2011). An automatic contour propagation method to follow parotid gland
deformation during head-and-neck cancer tomotherapy. Phys. Med. Biol..

[pmbaacb65bib015] Fallone B G, Murray B, Rathee S, Stanescu T, Steciw S, Vidakovic S, Blosser E, Tymofichuk D (2009). First MR images obtained during megavoltage photon irradiation
from a prototype integrated linac-MR system. Med. Phys..

[pmbaacb65bib016] Fritscher K D, Peroni M, Zaffino P, Spadea M F, Schubert R, Sharp G (2014). Automatic segmentation of head and neck CT images for
radiotherapy treatment planning using multiple atlases, statistical
appearance models, and geodesic active contours. Med. Phys..

[pmbaacb65bib017] Han X, Hoogeman M S, Levendag P C, Hibbard L S, Teguh D N, Voet P, Cowen A C, Wolf T K (2008). Atlas-based auto-segmentation of head and neck CT
images. MICCAI 2008.

[pmbaacb65bib018] Hissoiny S, Ozell B, Bouchard H, Després P (2011). GPUMCD: a new GPU-oriented Monte Carlo dose calculation
platform. Med. Phys..

[pmbaacb65bib019] Hoang Duc A K (2015). Validation of clinical acceptability of an atlas-based
segmentation algorithm for the delineation of organs at risk in head and
neck cancer. Med. Phys..

[pmbaacb65bib020] Hunt M A, Jackson A, Narayana A, Lee N (2006). Geometric factors influencing dosimetric sparing of the parotid
glands using IMRT. Int. J. Radiat. Oncol. Biol. Phys..

[pmbaacb65bib021] Köhler M, Vaara T, Grootel M V, Hoogeveen R, Kemppainen R, Renisch S (2015). MR-only simulation for radiotherapy planning treatment
planning. White Paper: Philips MRCAT for Prostate Dose Calculations Using only MRI
Data.

[pmbaacb65bib022] La Macchia M, Fellin F, Amichetti M, Cianchetti M, Gianolini S, Paola V, Lomax A J, Widesott L (2012). Systematic evaluation of three different commercial software
solutions for automatic segmentation for adaptive therapy in head-and-neck,
prostate and pleural cancer. Radiat. Oncol..

[pmbaacb65bib023] Lagendijk J J, Raaymakers B W, Van Den Berg C A, Moerland M A, Philippens M E, Van Vulpen M (2014). MR guidance in radiotherapy. Phys. Med. Biol..

[pmbaacb65bib024] Liney G P (2016). Technical Note: experimental results from a prototype high-field
inline MRI-linac. Med. Phys..

[pmbaacb65bib025] Metcalfe P, Liney G P, Holloway L, Walker A, Barton M, Delaney G P, Vinod S, Tomé W (2013). The potential for an enhanced role for MRI in radiation-therapy
treatment planning. Technol. Cancer Res. Treat..

[pmbaacb65bib026] Modat M, Cash D M, Daga P, Winston G P, Duncan J S, Ourselin S (2014). Global image registration using a symmetric block-matching
approach. J. Med. Imaging.

[pmbaacb65bib027] Modat M, Ridgway G R, Taylor Z A, Lehmann M, Barnes J, Hawkes D J, Fox N C, Ourselin S (2010). Fast free-form deformation using graphics processing
units. Comput. Methods Prog. Biomed..

[pmbaacb65bib028] Mutic S, Dempsey J F (2014). The ViewRay system: magnetic resonance-guided and controlled
radiotherapy. Semin. Radiat. Oncol..

[pmbaacb65bib029] Nelms B E, Tomé W A, Robinson G, Wheeler J (2012). Variations in the contouring of organs at risk: test case from a
patient with oropharyngeal cancer. Int. J. Radiat. Oncol. Biol. Phys..

[pmbaacb65bib030] Nyholm T, Jonsson J (2014). Counterpoint: opportunities and challenges of a magnetic
resonance imaging-only radiotherapy work flow. Semin. Radiat. Oncol..

[pmbaacb65bib031] Pekar V, Allaire S, Qazi A (2010). Head and neck auto-segmentation challenge: segmentation of the
parotid glands. MICCAI 2010: a Grand Challenge for the Clinic (August).

[pmbaacb65bib032] Qazi A A, Pekar V, Kim J, Xie J, Breen S L, Jaffray D A (2011). Auto-segmentation of normal and target structures in head and
neck CT images: a feature-driven model-based approach. Med. Phys..

[pmbaacb65bib033] Raaymakers B W (2009). Integrating a 1.5 T MRI scanner with a 6 MV accelerator: proof of
concept. Phys. Med. Biol..

[pmbaacb65bib034] Rasch C R, Steenbakkers R J, Fitton I, Duppen J C, Nowak P J, Pameijer F A, Eisbruch A, Kaanders J H, Paulsen F, van Herk M (2010). Decreased 3D observer variation with matched CT-MRI, for target
delineation in Nasopharynx cancer. Radiat. Oncol..

[pmbaacb65bib035] Schmidt M A, Payne G S (2015). Radiotherapy planning using MRI. Phys. Med. Biol..

[pmbaacb65bib036] Sims R (2009). A pre-clinical assessment of an atlas-based automatic
segmentation tool for the head and neck. Radiother. Oncol..

[pmbaacb65bib037] Spearman C (1904). Spearman’s rank correlation coefficient. Am. J. Psychol..

[pmbaacb65bib038] Student (1908). The probable error of a mean. Biometrika.

[pmbaacb65bib039] Sun Y (2014). Recommendation for a contouring method and atlas of organs at
risk in nasopharyngeal carcinoma patients receiving intensity-modulated
radiotherapy. Radiother. Oncol..

[pmbaacb65bib040] Teguh D N (2011). Clinical validation of atlas-based auto-segmentation of multiple
target volumes and normal tissue (swallowing/mastication) structures in the
head and neck. Int. J. Radiat. Oncol. Biol Phys..

[pmbaacb65bib041] Tsuji S Y, Hwang A, Weinberg V, Yom S S, Quivey J M, Xia P (2010). Dosimetric evaluation of automatic segmentation for adaptive IMRT
for head-and-neck cancer. Int. J. Radiat. Oncol. Biol. Phys..

[pmbaacb65bib042] Van Leemput K, Maes F, Vandermeulen D, Suetens P (1999). Automated model-based bias field correction of MR images of the
brain. IEEE Trans. Med. Imaging.

[pmbaacb65bib043] Veeraraghavan H, Tyagi N, Hunt M, Lee N, Deasy J (2015). SU-F-303-16: Multi-atlas and learning based segmentation of head
and neck normal structures from multi-parametric MRI. Med. Phys..

[pmbaacb65bib044] Vinod S K, Jameson M G, Min M, Holloway L C (2016). Uncertainties in volume delineation in radiation oncology: a
systematic review and recommendations for future studies. Radiother. Oncol..

[pmbaacb65bib045] Voet P W J, Dirkx M L P, Teguh D N, Hoogeman M S, Levendag P C, Heijmen B J M (2011). Does atlas-based autosegmentation of neck levels require
subsequent manual contour editing to avoid risk of severe target
underdosage? A dosimetric analysis. Radiother. Oncol..

[pmbaacb65bib046] Wardman K, Prestwich R J D, Gooding M J, Speight R J (2016). The feasibility of atlas-based automatic segmentation of MRI for
H&N radiotherapy planning. J. Appl. Clin. Med. Phys..

[pmbaacb65bib047] Warfield S K, Zou K H, Wells W M (2004). Simultaneous truth and performance level estimation (STAPLE): an
algorithm for the validation of image segmentation. IEEE Trans. Med. Imaging.

[pmbaacb65bib048] Welsh L (2015). Prospective, longitudinal, multi-modal functional imaging for
radical chemo-IMRT treatment of locally advanced head and neck cancer: the
INSIGHT study. Radiat. Oncol..

[pmbaacb65bib049] Yang X, Wu N, Cheng G, Zhou Z, Yu D S, Beitler J J, Curran W J, Liu T (2014). Automated segmentation of the parotid gland based on atlas
registration and machine learning: a longitudinal MRI study in head-and-neck
radiation therapy. Int. J. Radiat. Oncol. Biol. Phys..

